# Supplementary material for: Antifungal Activity of Chemical Constituents from Piper pesaresanum C. DC. and Derivatives against Phytopathogen Fungi of Cocoa
Source: Molecules. 2021 May 28;26(11):3256. doi: 10.3390/molecules26113256 (PMC8198927; doi:10.3390/molecules26113256)
Supplement: Supplementary file 1 [file molecules-26-03256-s001.zip › molecules-1200879-supplementary materials_v4-send-3-molecules.pdf]

Supplementary Materials

Antifungal Activity of Chemical Constituents from *Piper pesaresanum* C. DC. and Derivatives Against Phytopathogen Fungi of Cocoa

Luis C. Chitiva-Chitiva [1], Cristóbal Ladino-Vargas [2], Luis E. Cuca-Suárez [1], Juliet A. Prieto-Rodríguez [2], Oscar J. Patiño-Ladino [1,*]

[1] Department of Chemistry, Faculty of Sciences, Universidad Nacional de Colombia, Sede Bogotá, 111321 Bogotá, Colombia; lchitiva@unal.edu.co (L.C.C.-C.); lecucas@unal.edu.co (L.E.C.-S.)

[2] Department of Chemistry, Faculty of Sciences, Pontificia Universidad Javeriana, Sede Bogotá, 110231 Bogotá, Colombia; cristoballadino@javeriana.edu.co (C.L.-V.); juliet.prieto@javeriana.edu.co (J.A.P.-R.)

* Correspondence: ojpatinol@unal.edu.co (O.J.P.-L); Tel.: +57-1-3165000 (ext. 14485)

Table of Contents:

Scheme S1. Diagram of the phytochemical study carried out on aerial part from *Piper pesaresanum*.

Figure S1. ^{1}H-NMR spectrum of 4-methoxynervogenic acid (**1**).

Figure S2. APT spectrum of 4-methoxynervogenic acid (**1**).

Figure S3. ^{1}H-NMR spectrum of nervogenic acid (**2**).

Figure S4. APT spectrum of nervogenic acid (**2**).

Figure S5. ^{1}H-NMR spectrum of 3-(3′,3′-dimethylallyl-1′-oxo)-5-(3′′,3′′-dimethylallyl)-4-hydroxybenzoic acid (**3**).

Figure S6. APT spectrum of 3-(3′,3′-dimethylallyl-1′-oxo)-5-(3′′,3′′-dimethylallyl)-4-hydroxybenzoic acid (**3**).

Figure S7. ^{1}H-NMR spectrum of 2′,6′-dihydroxy-4′-methoxydihydrochalcone (**4**).

Figure S8. APT spectrum of 2′,6′-dihydroxy-4′-methoxydihydrochalcone (**4**).

Figure S9. ^{1}H-NMR spectrum of methyl 3,5-bis(3′,3′-dimethylallyl)-4-methoxybenzoate (**8**).

Figure S10. APT spectrum of methyl 3,5-bis(3′,3′-dimethylallyl)-4-methoxybenzoate (**8**).

Figure S11. ^{1}H-NMR spectrum of 2,6-bis(3',3'-dimethylallyl)-1-methoxybenzene (**9**).

Figure S12. APT spectrum of 2,6-bis(3',3'-dimethylallyl)-1-methoxybenzene (**9**).

Figure S13. ^{1}H-NMR spectrum of 3,5-diisopentyl-4-methoxybenzoic acid (**10**).

Figure S14. APT spectrum of 3,5-diisopentyl-4-methoxybenzoic acid (**10**).

Figure S15. ^{1}H-NMR spectrum of 2,2-dimethyl-8-(3′,3′-dimethylallyl)-2*H*-1-chromene-6-carboxylic acid (**11**).

Figure S16. APT spectrum of 2,2-dimethyl-8-(3′,3′-dimethylallyl)-2*H*-1-chromene-6-carboxylic acid (**11**).

Figure S17. ^{1}H-NMR spectrum of 2,2-dimethyl-8-(3',3'-dimethylallyl)-4-oxochroman-6-carboxylic acid (**12**).

Figure S18. APT spectrum of 2,2-dimethyl-8-(3',3'-dimethylallyl)-4-oxochroman-6-carboxylic acid (**12**).

Figure S19. ^{1}H-NMR spectrum of 2´,4´,6´-trimethoxydihydrochalcone (**13**).

Figure S20. APT spectrum of 2´,4´,6´-trimethoxydihydrochalcone (**13**).

Figure S21. ^{1}H-NMR spectrum of 1-(2′,6′-dihydroxy-4′-methoxyphenyl)-3-phenylpropane (**14**).

Figure S22. APT spectrum of 1-(2′,6′-dihydroxy-4′-methoxyphenyl)-3-phenylpropane (**14**).

Figure S23. HRESIMS spectrum of 2,6-bis(3′,3′-dimetilalil)-1-metoxibenceno (**9**).

Figure S24. HRESIMS spectrum of 3,5-diisopentyl-4-methoxybenzoic acid (**10**).

Figure S25. HRESIMS spectrum of 2,2-dimethyl-8-(3',3'-dimethylallyl)-4-oxochroman-6-carboxylic acid (**12**).

Figure S26. HRESIMS spectrum of 1-(2′,6′-dihydroxy-4′-methoxyphenyl)-3-phenylpropane (**14**).

Figure S27. Total ionic current chromatogram (TIC) of the sterol mixture.

Figure S28. Mass spectrum campesterol (**5**).

Figure S29. Mass spectrum of stigmasterol (**6**).

Figure S30. Mass spectrum of γ-sitosterol (**7**).

Figure S31. Retention times of *n*-paraffins (C_{10}-C_{35}) analyzed under the same chromatographic conditions as the samples were used.

Table S1. Retention indices of Kovats (KIs) experimental and reported in the literature for the mixture of sterols.

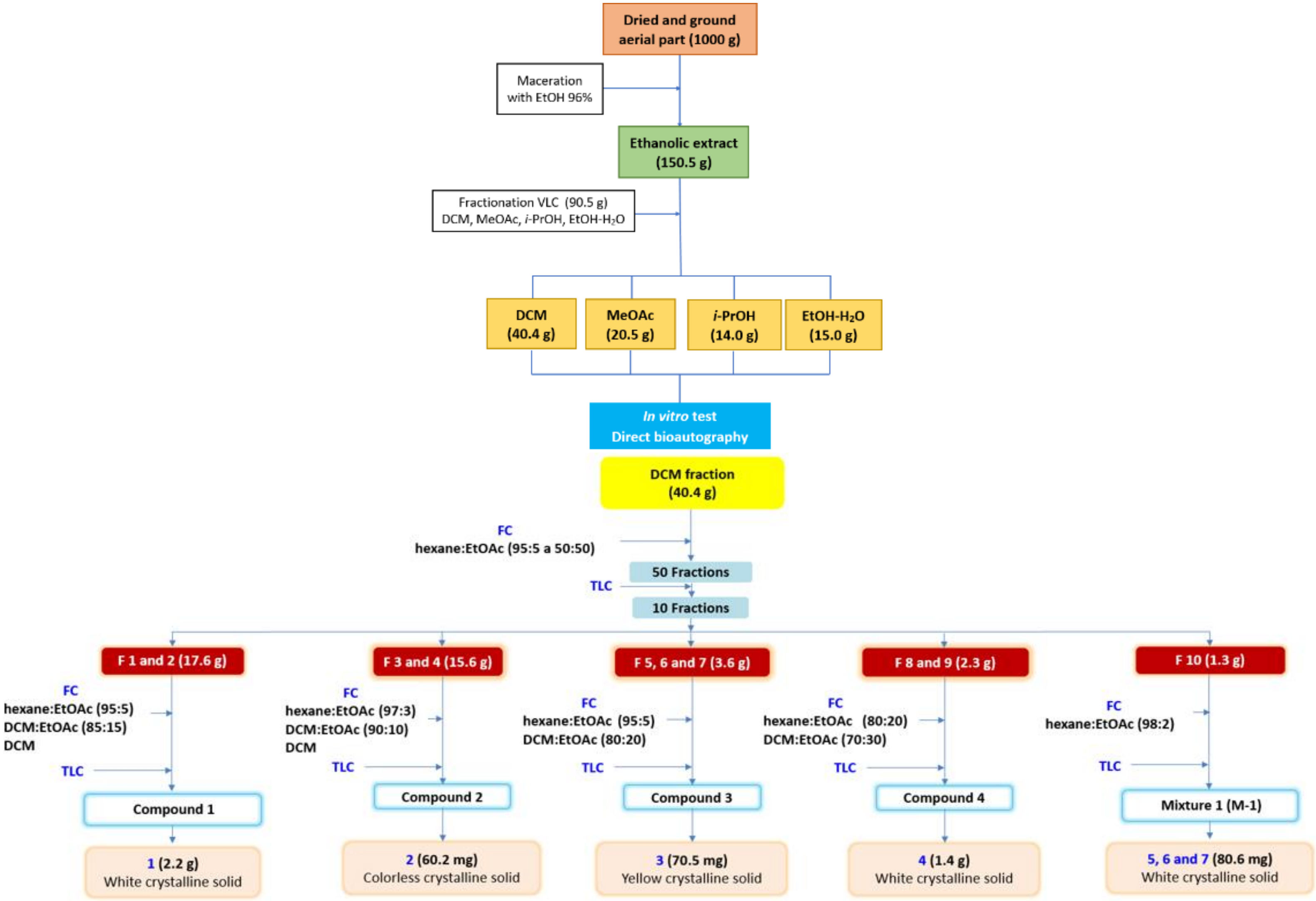

Scheme S1. Diagram of the phytochemical study carried out on aerial part from *Piper pesaresanum*.

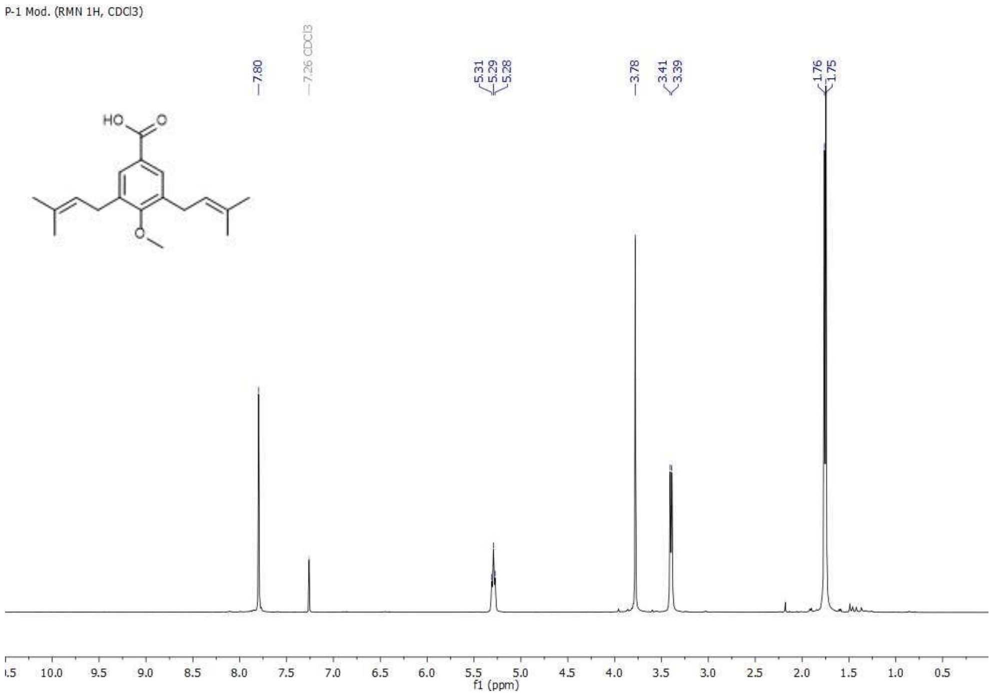

Figure S1. ^{1}H-NMR spectrum of 4-methoxynervogenic acid (**1**).

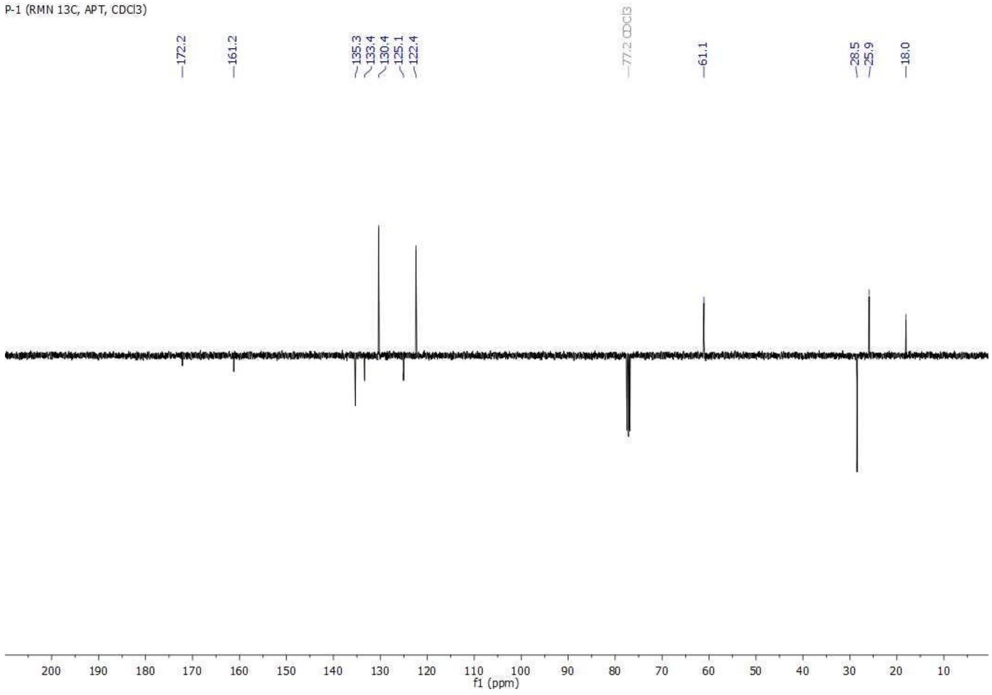

Figure S2. APT spectrum of 4-methoxynervogenic acid (**1**).

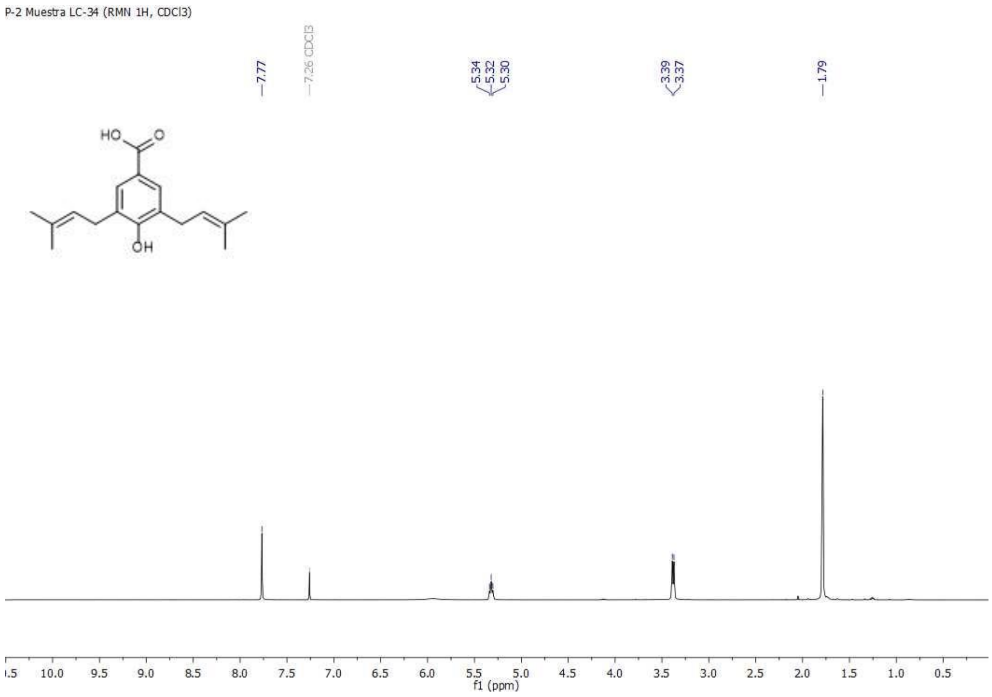

Figure S3. ^{1}H-NMR spectrum of nervogenic acid (**2**).

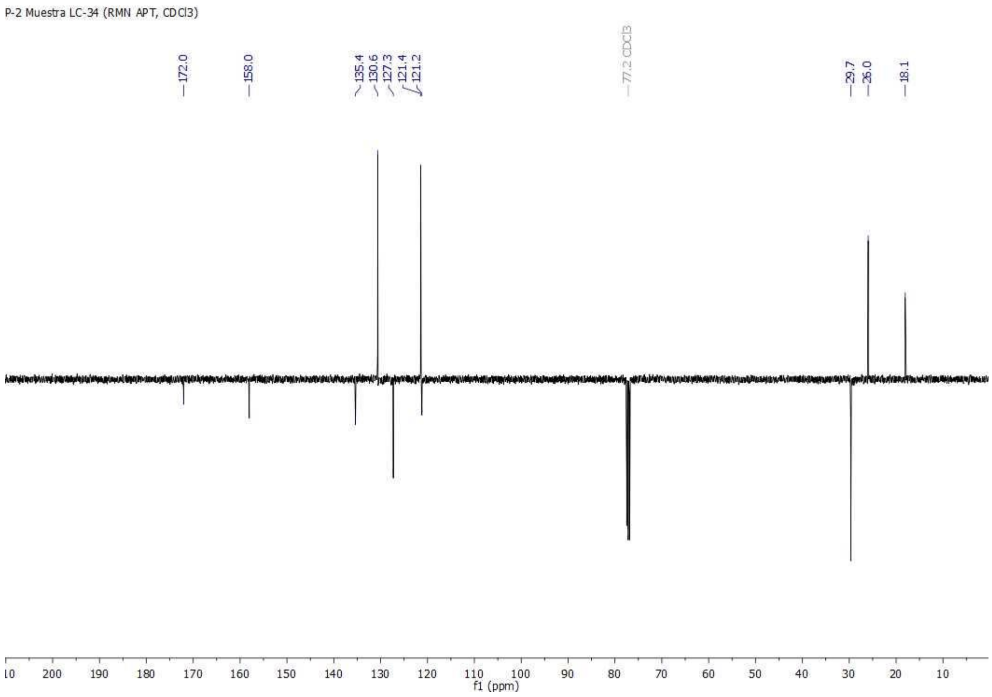

Figure S4. APT spectrum of nervogenic acid (**2**).

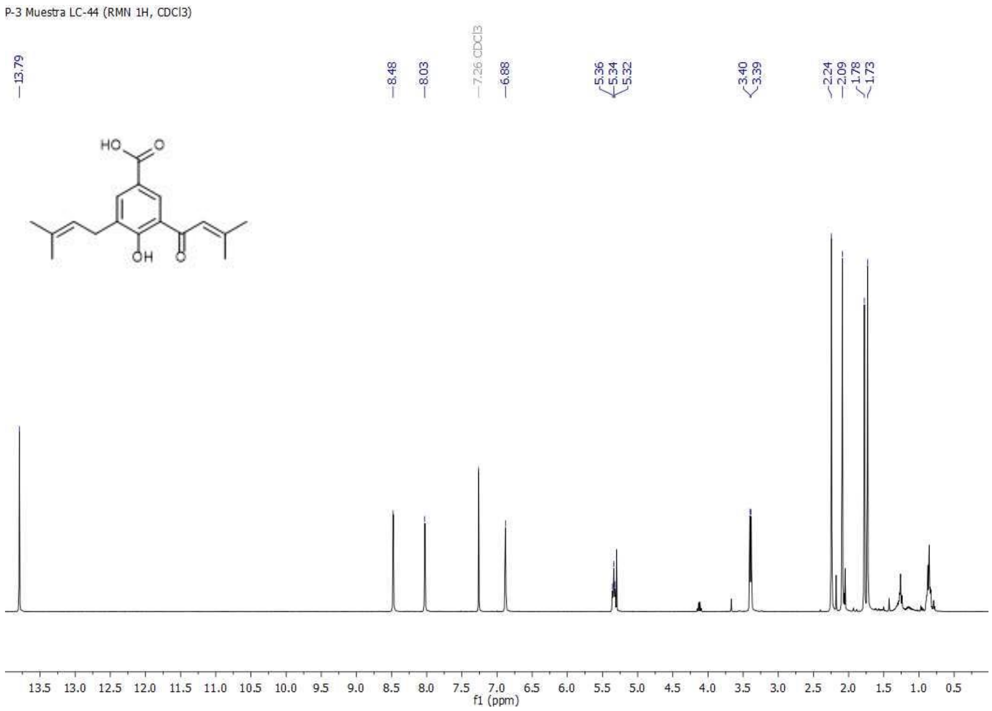

Figure S5. ^{1}H-NMR spectrum of 3-(3′,3′-dimethylallyl-1′-oxo)-5-(3′′,3′′-dimethylallyl)-4-hydroxybenzoic acid (**3**).

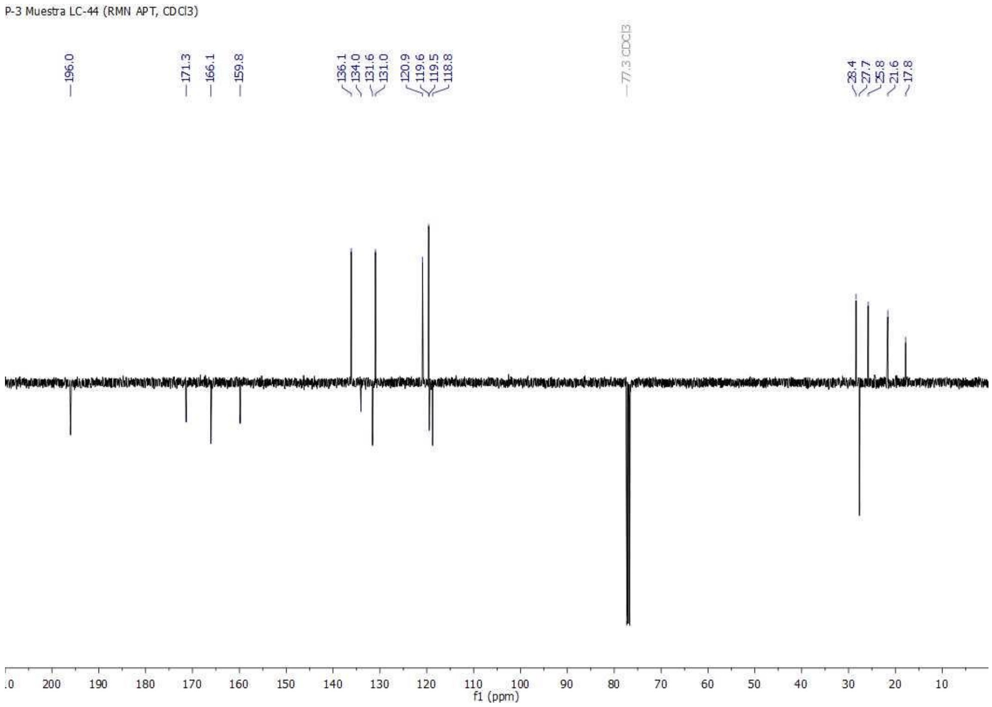

Figure S6. APT spectrum of 3-(3′,3′-dimethylallyl-1′-oxo)-5-(3′′,3′′-dimethylallyl)-4-hydroxybenzoic acid (**3**).

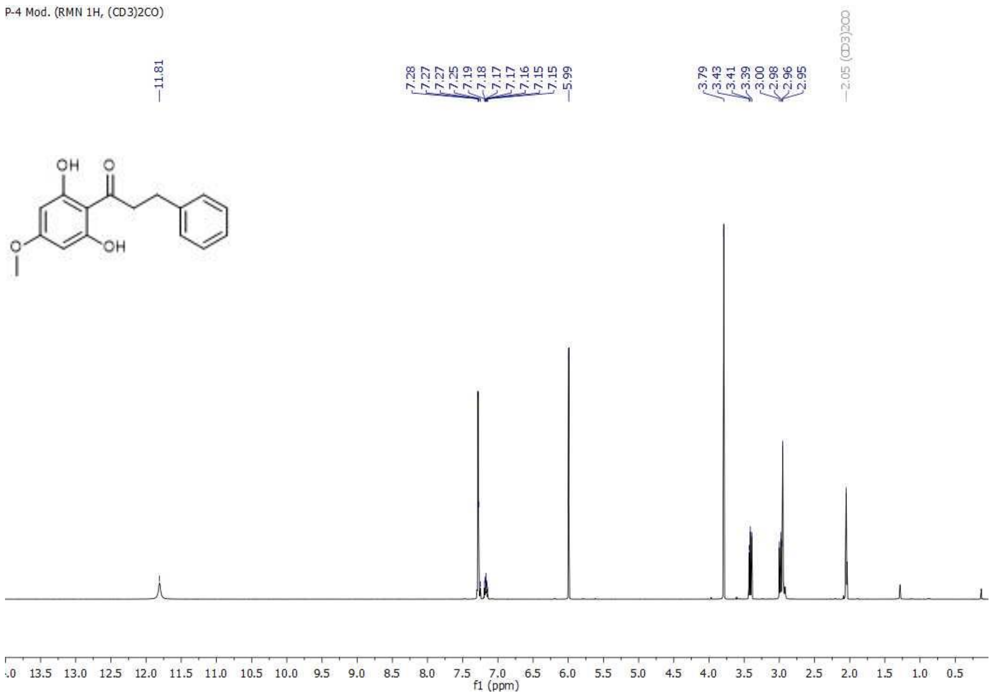

Figure S7. ^{1}H-NMR spectrum of 2′,6′-dihydroxy-4′-methoxydihydrochalcone (**4**).

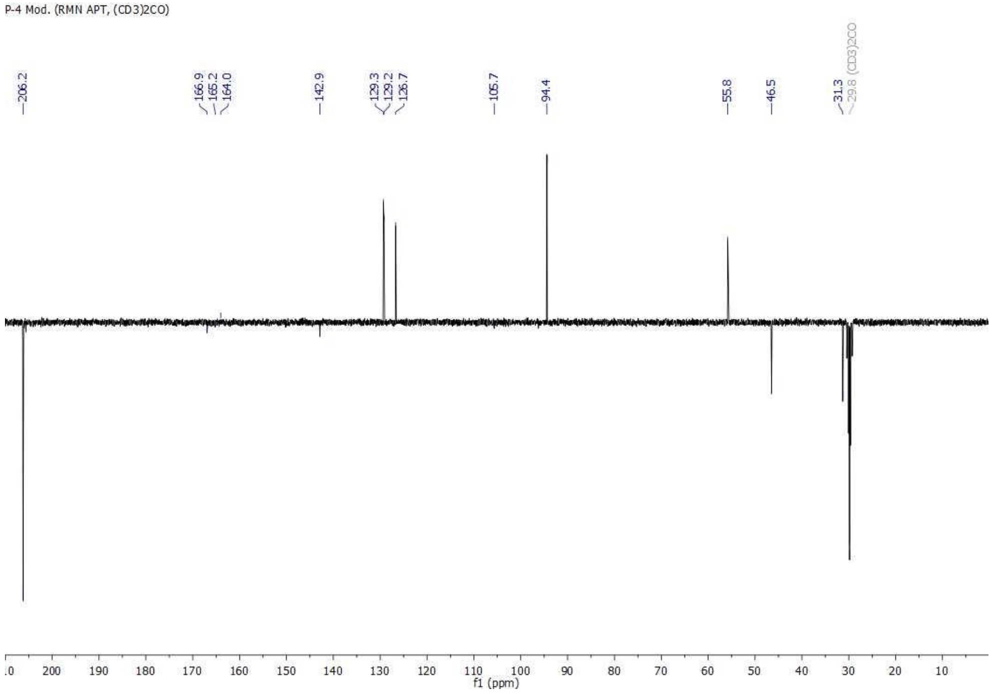

Figure S8. APT spectrum of 2′,6′-dihydroxy-4′-methoxydihydrochalcone (**4**).

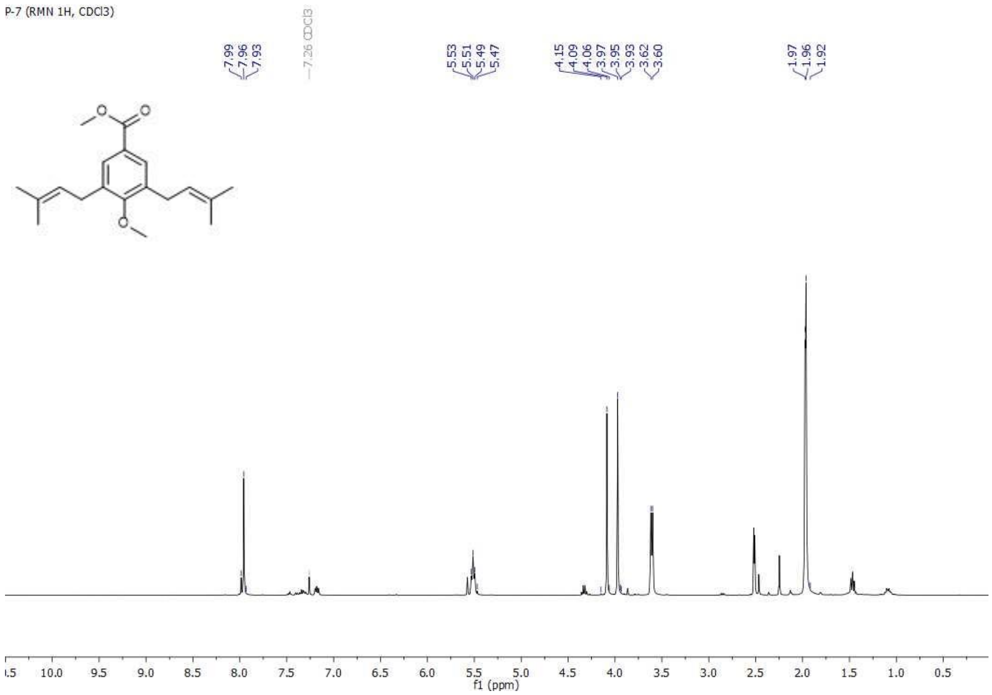

Figure S9. ^{1}H-NMR spectrum of methyl 3,5-bis(3′,3′-dimethylallyl)-4-methoxybenzoate (**8**).

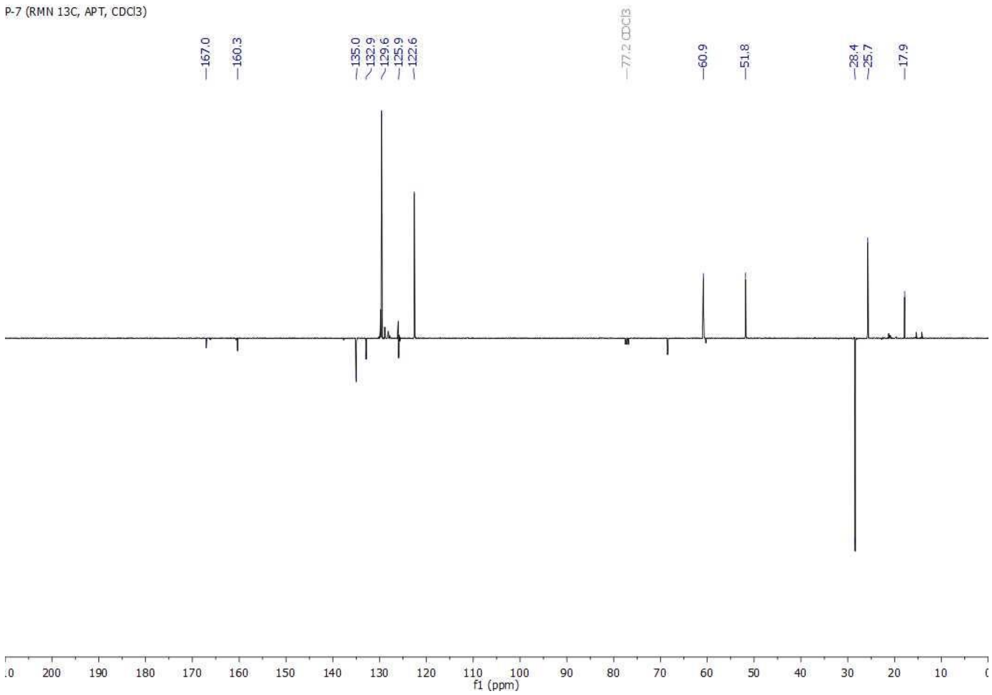

Figure S10. APT spectrum of methyl 3,5-bis(3′,3′-dimethylallyl)-4-methoxybenzoate (**8**).

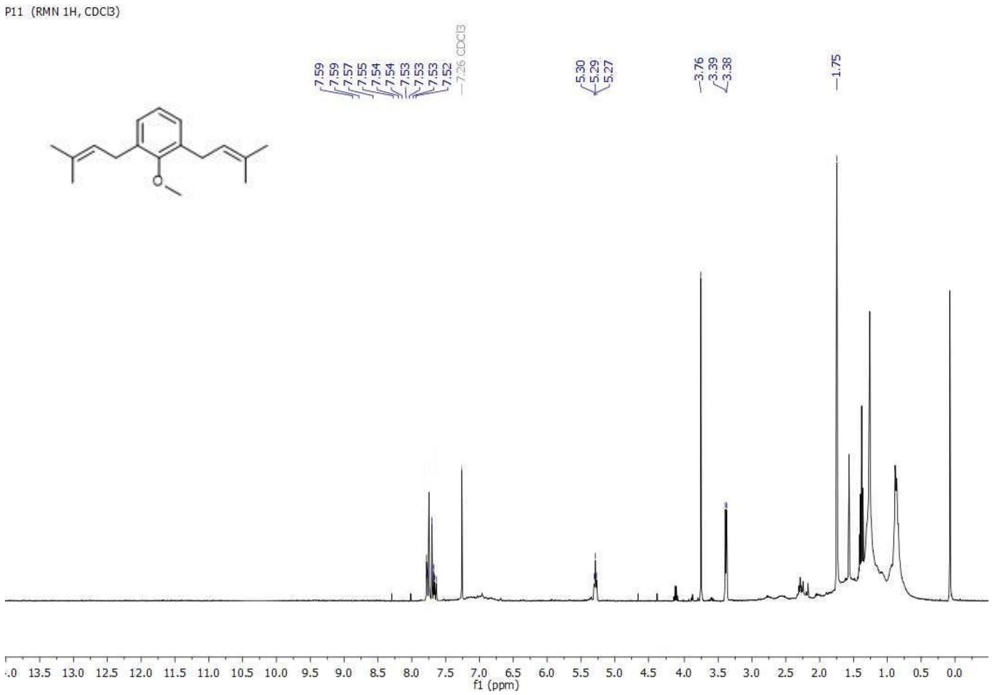

Figure S11. ^{1}H-NMR spectrum of 2,6-bis(3',3'-dimethylallyl)-1-methoxybenzene (**9**).

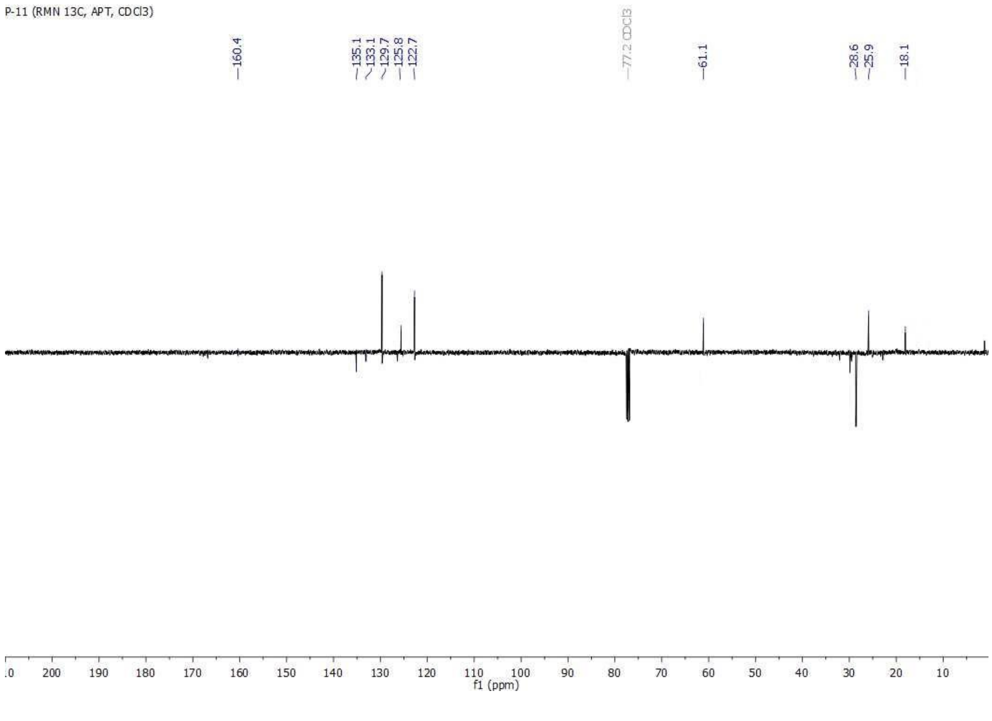

Figure S12. APT spectrum of 2,6-bis(3',3'-dimethylallyl)-1-methoxybenzene (**9**).

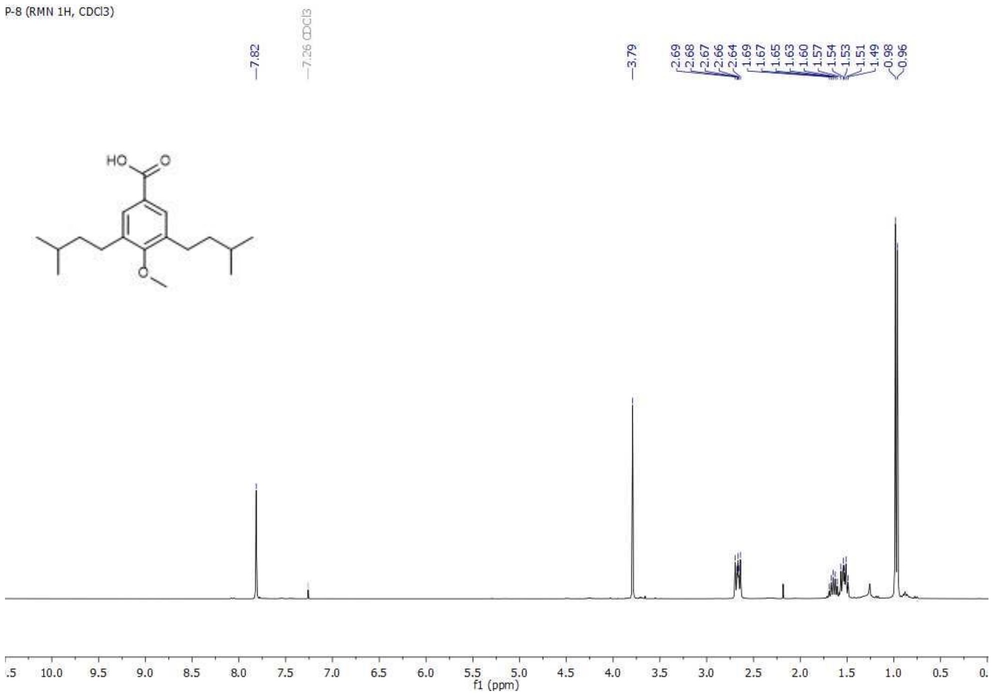

Figure S13. ^{1}H-NMR spectrum of 3,5-diisopentyl-4-methoxybenzoic acid (**10**).

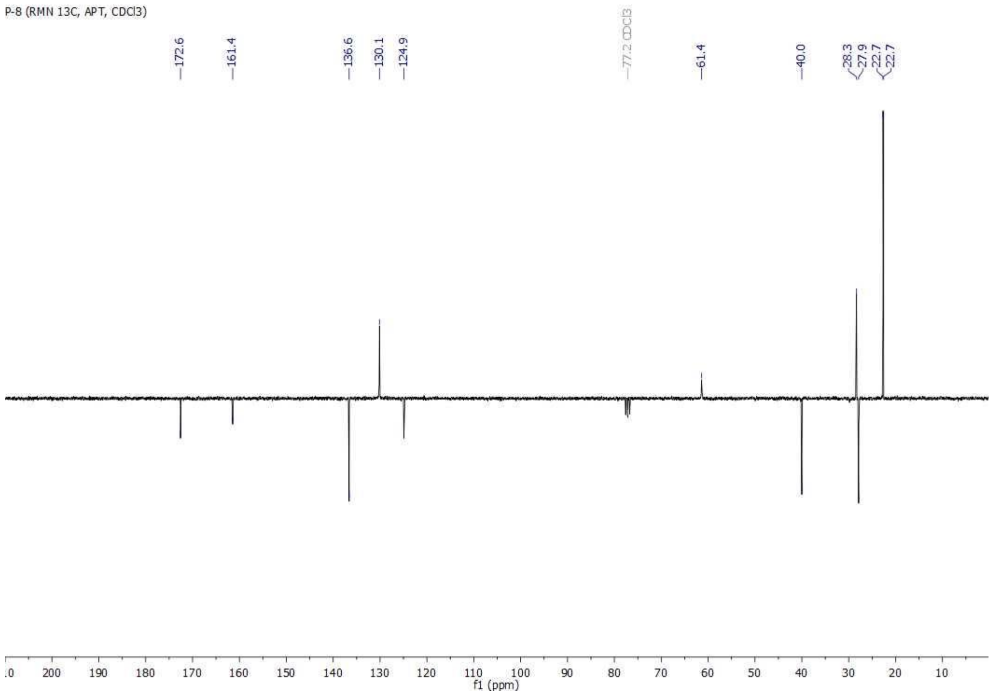

Figure S14. APT spectrum of 3,5-diisopentyl-4-methoxybenzoic acid (**10**).

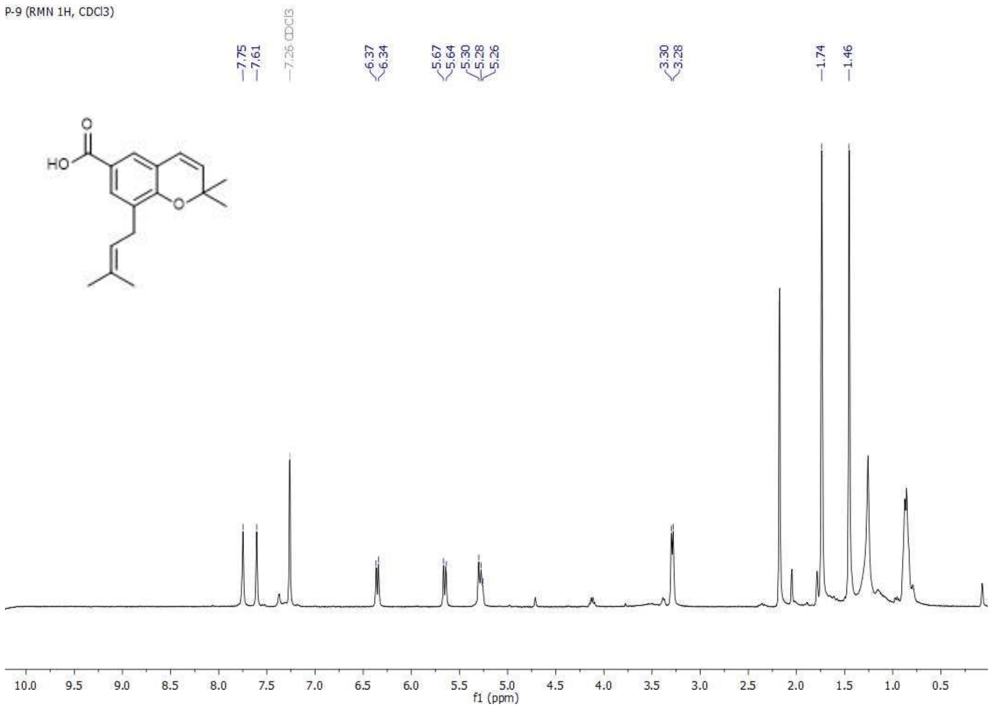

Figure S15. ^{1}H-NMR spectrum of 2,2-dimethyl-8-(3′,3′-dimethylallyl)-2*H*-1-chromene-6-carboxylic acid (**11**).

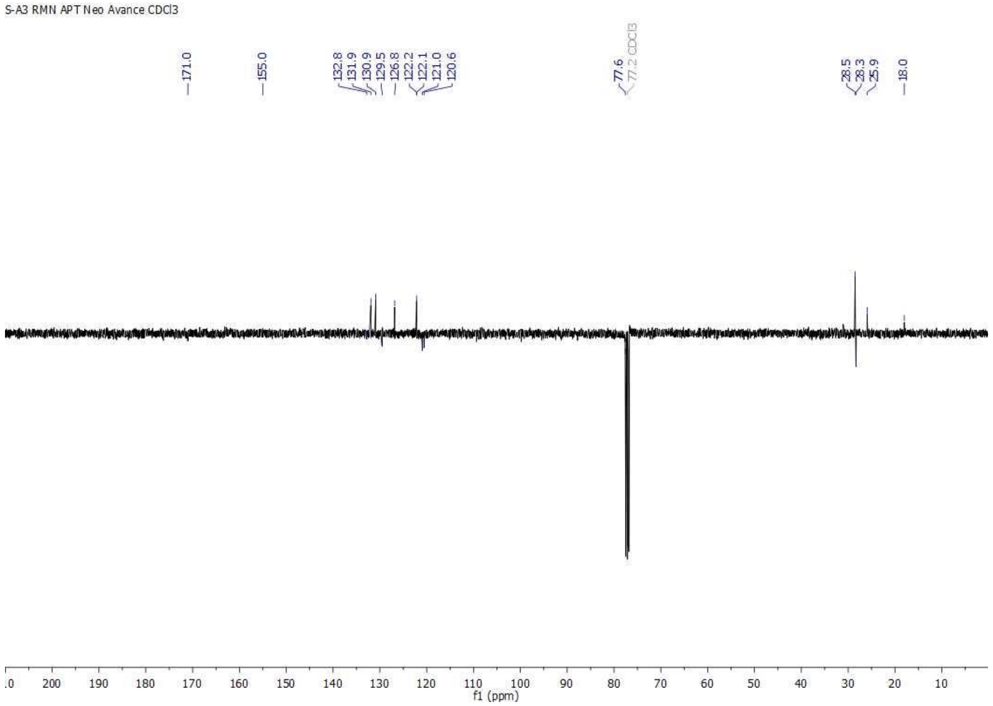

Figure S16. APT spectrum of 2,2-dimethyl-8-(3′,3′-dimethylallyl)-2*H*-1-chromene-6-carboxylic acid (**11**).

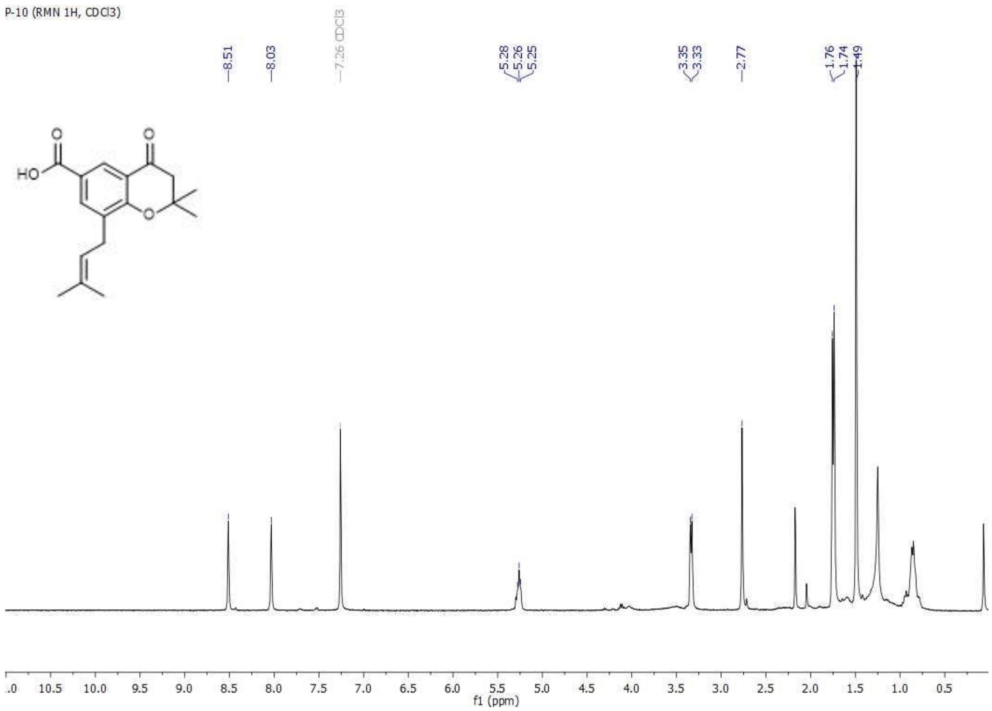

Figure S17. ^{1}H-NMR spectrum of 2,2-dimethyl-8-(3',3'-dimethylallyl)-4-oxochroman-6-carboxylic acid (**12**).

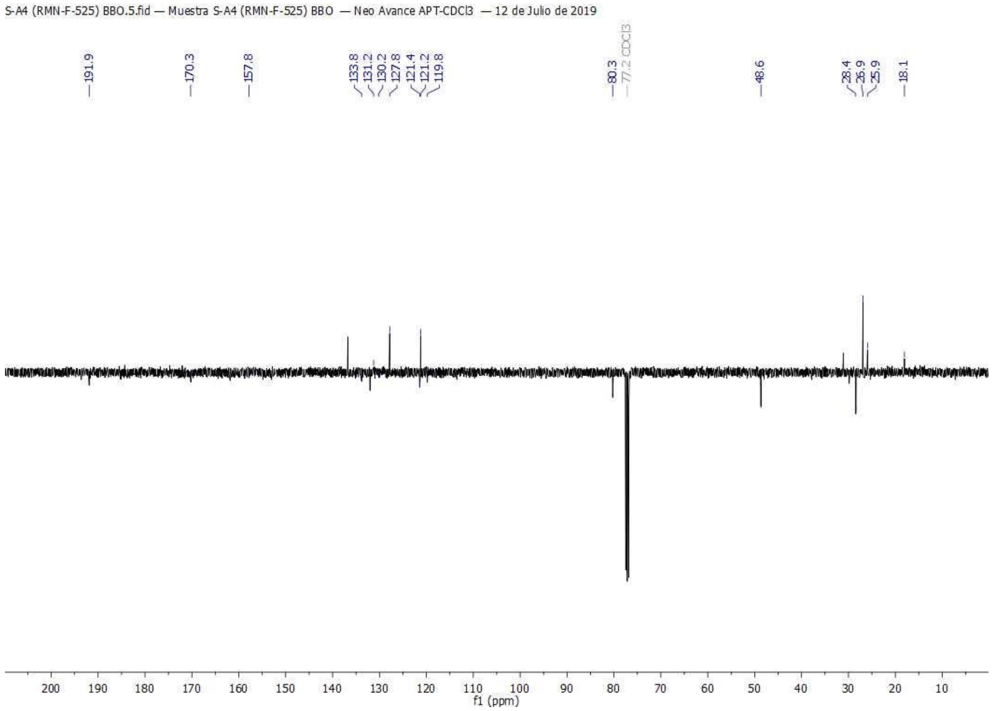

Figure S18. APT spectrum of 2,2-dimethyl-8-(3',3'-dimethylallyl)-4-oxochroman-6-carboxylic acid (**12**).

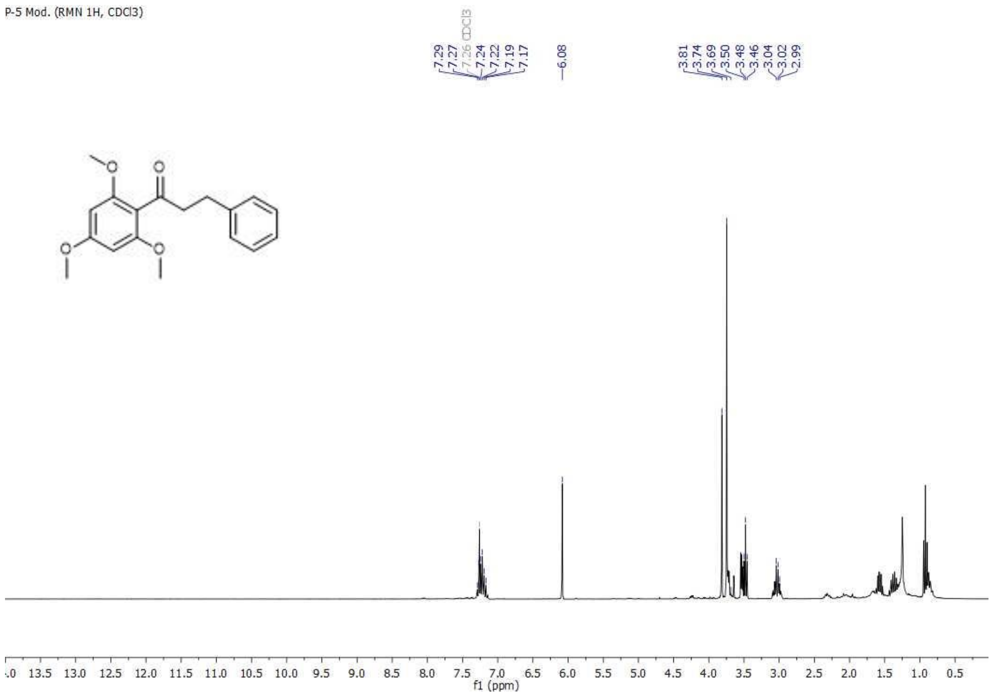

Figure S19. ^{1}H-NMR spectrum of 2′,4′,6′-trimethoxydihydrochalcone (**13**).

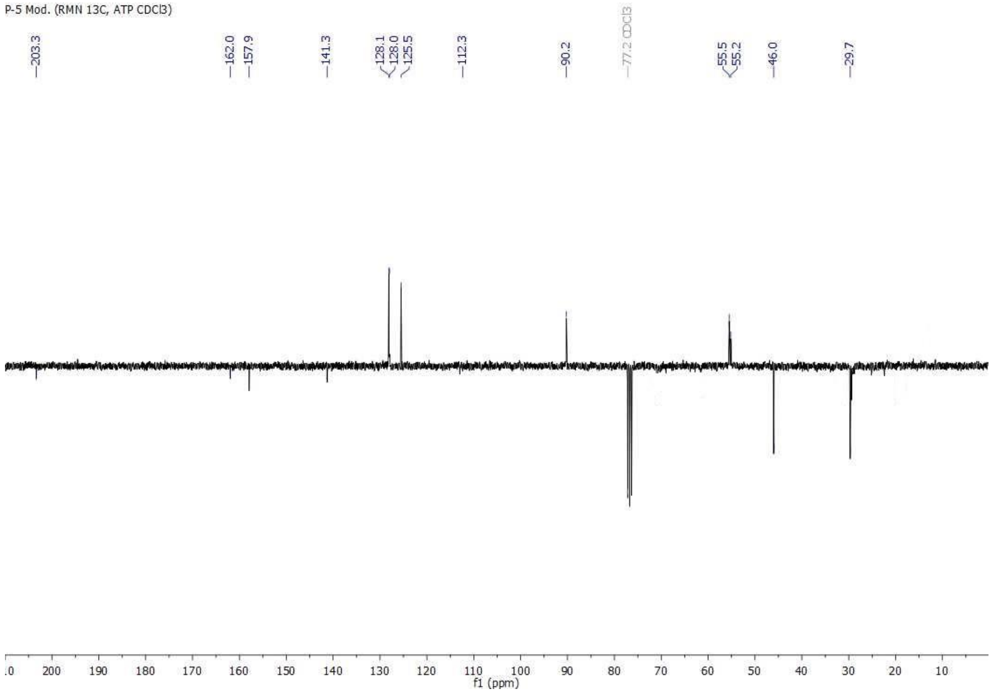

Figure S20. APT spectrum of 2′,4′,6′-trimethoxydihydrochalcone (**13**).

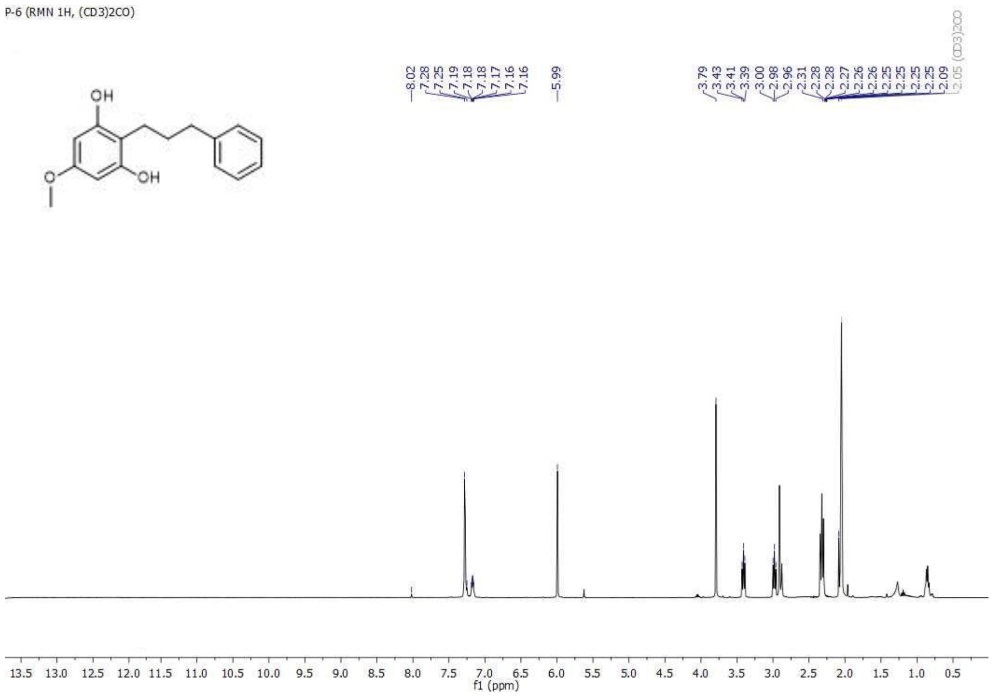

Figure S21. ^{1}H-NMR spectrum of 1-(2′,6′-dihydroxy-4′-methoxyphenyl)-3-phenylpropane (**14**).

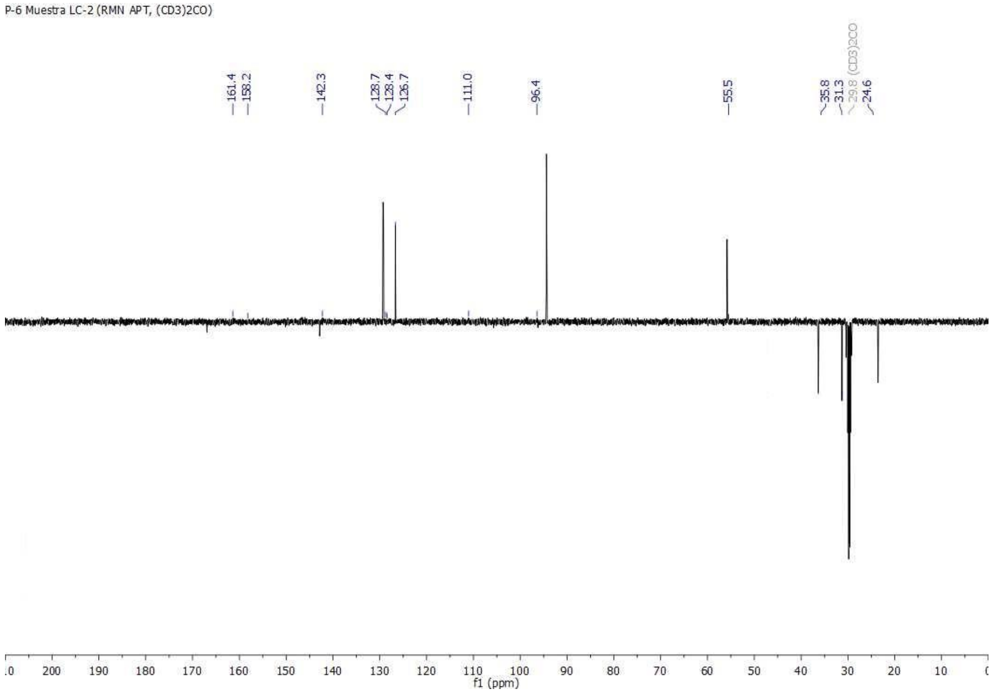

Figure S22. APT spectrum of 1-(2′,6′-dihydroxy-4′-methoxyphenyl)-3-phenylpropane (**14**).

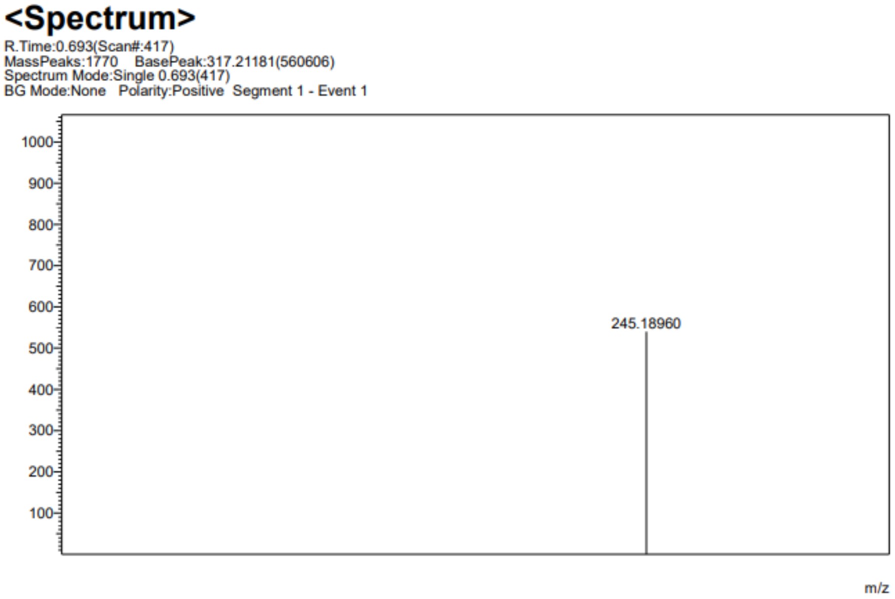

Figure S23. HRESIMS spectrum of 2,6-bis(3′,3′-dimetilalil)-1-metoxibenceno (**9**).

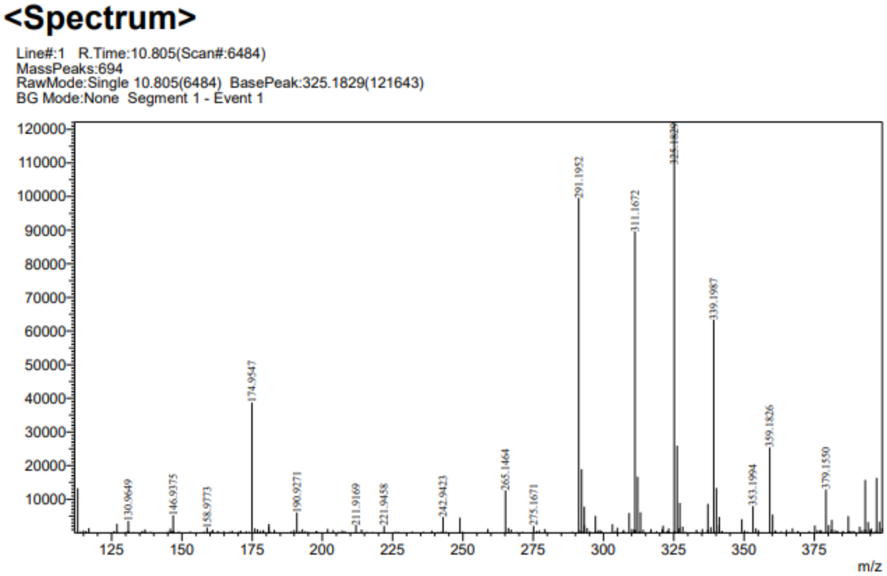

Figure S24. HRESIMS spectrum of 3,5-diisopentyl-4-methoxybenzoic acid (**10**).

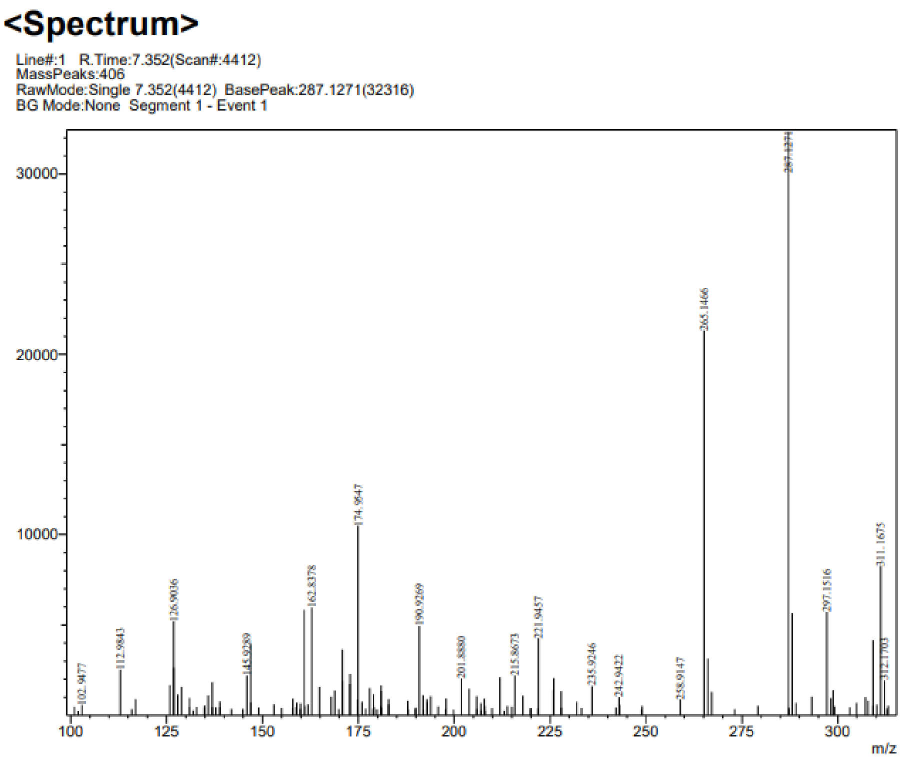

Figure S25. HRESIMS spectrum of 2,2-dimethyl-8-(3',3'-dimethylallyl)-4-oxochroman-6-carboxylic acid (**12**).

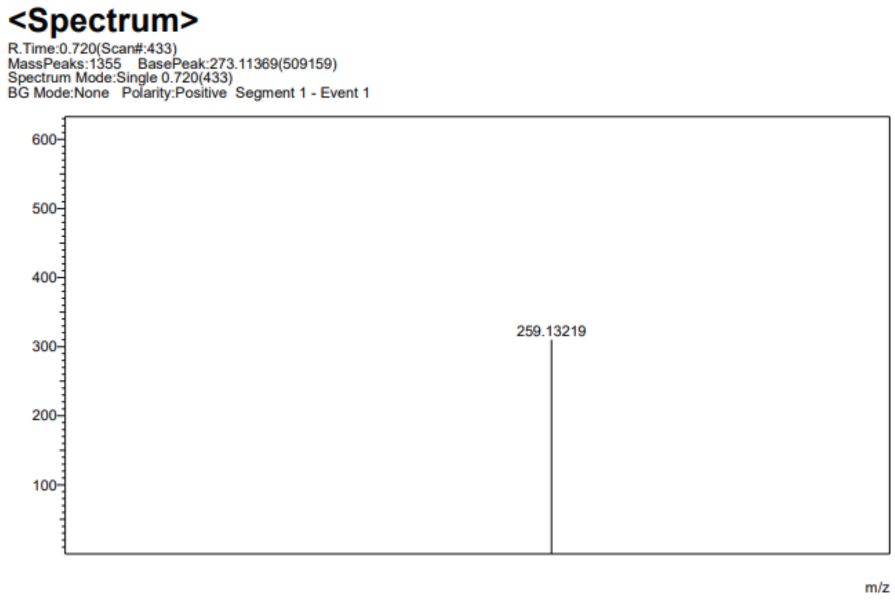

Figure S26. HRESIMS spectrum of 1-(2′,6′-dihydroxy-4′-methoxyphenyl)-3-phenylpropane (**14**).

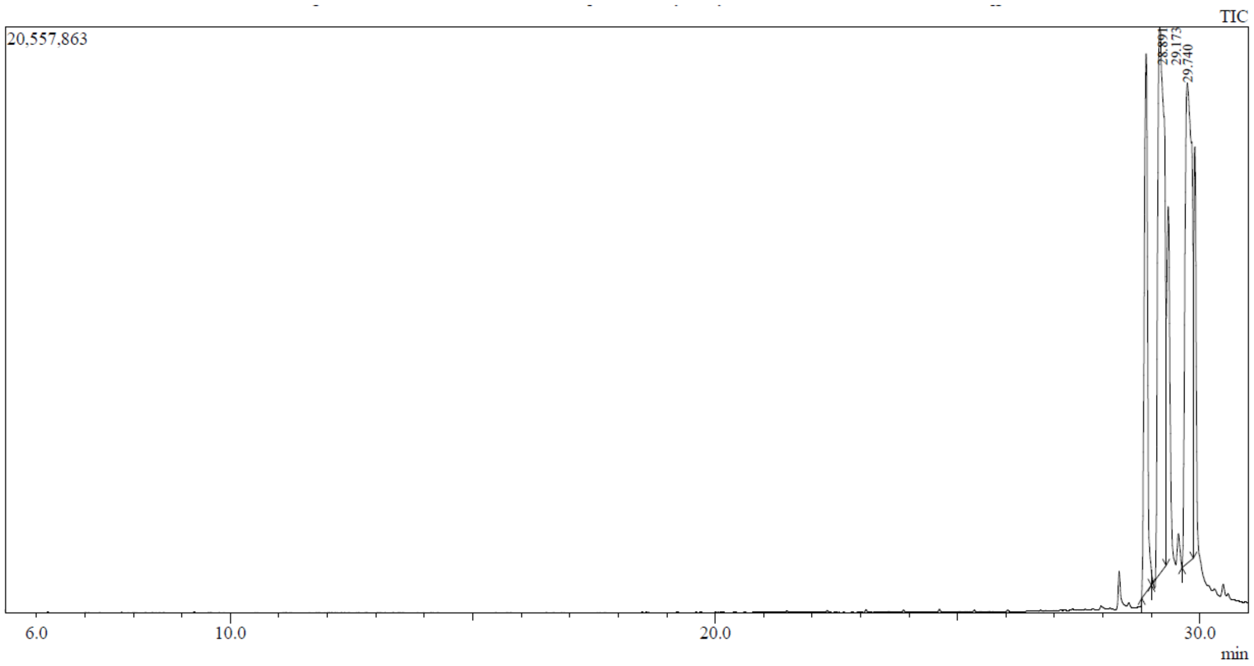

Peak Report TIC

Peak#	R.Time	I.Time	F.Time	Area	Area%	Height	Height%	A/H	Mark	Name
1	28.891	28.800	29.000	89331666	20.09	18879872	34.40	4.73		Campesterol
2	29.173	29.000	29.300	183130846	41.19	19162586	34.91	9.56	V	Stigmasterol
3	29.740	29.633	29.867	172171907	38.72	16841256	30.69	10.22		.gamma.-Sitosterol
				444634419	100.00	54883714	100.00			

Figure S27. Total ionic current chromatogram (TIC) of the sterol mixture.

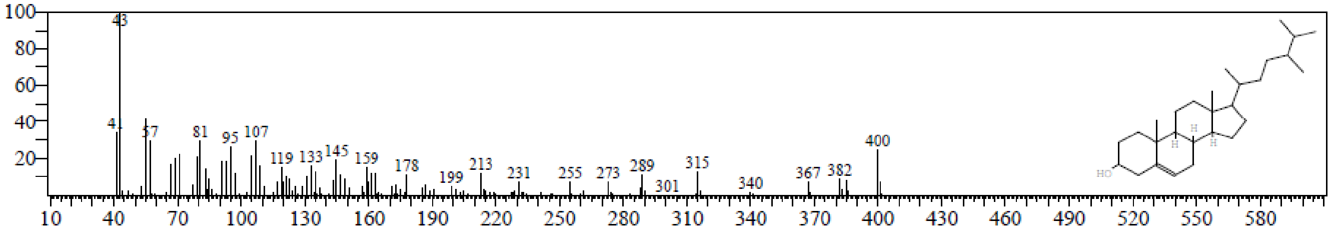

Figure S28. Mass spectrum campesterol (**5**).

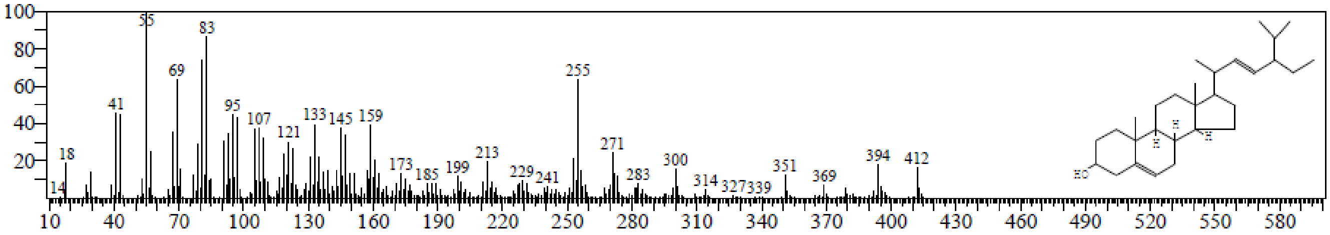

Figure S29. Mass spectrum of stigmasterol (**6**).

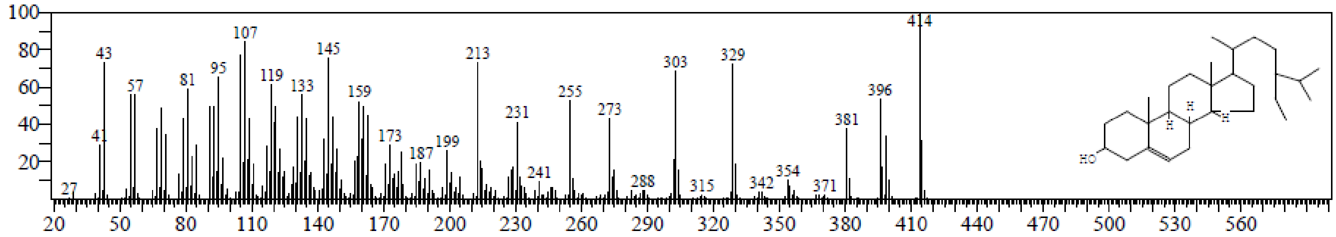

Figure S30. Mass spectrum of γ-sitosterol (**7**).

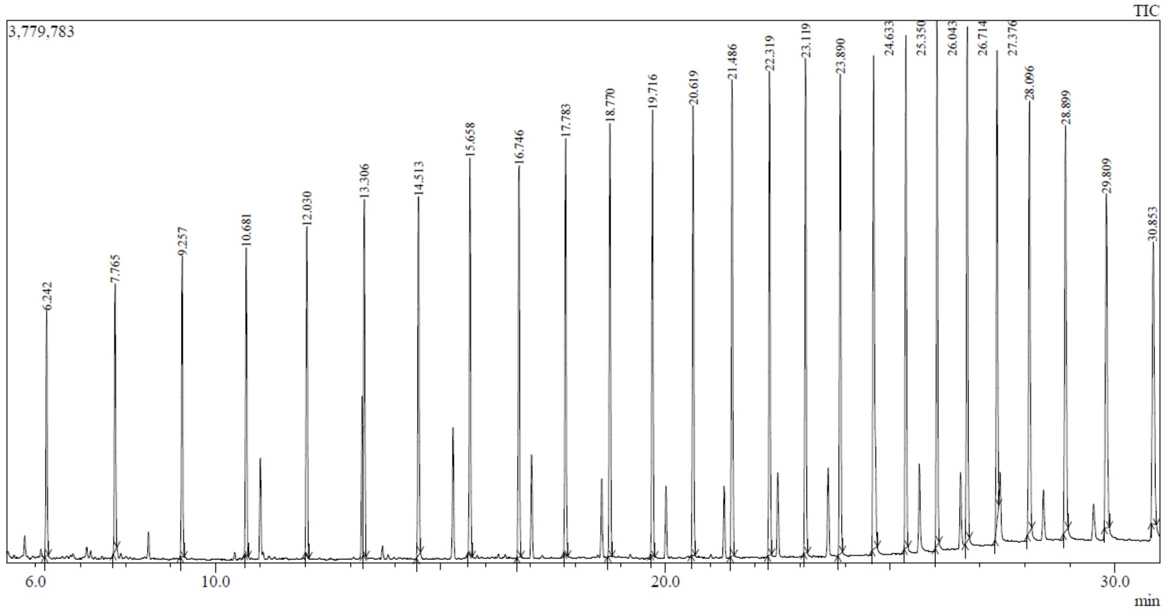

Peak Report TIC

Peak#	R.Time	I.Time	F.Time	Area	Area%	Height	Height%	A/H	Mark	Name
1	6.242	6.200	6.283	3410286	2.11	1706863	2.31	2.00		Decane
2	7.765	7.725	7.808	3742555	2.32	1859724	2.52	2.01		Undecane
3	9.257	9.217	9.300	4208803	2.61	2090729	2.83	2.01		Dodecane
4	10.681	10.642	10.725	4362090	2.70	2141663	2.90	2.04		Tridecane
5	12.030	11.992	12.075	4769689	2.96	2288692	3.10	2.08		Tetradecane
6	13.306	13.283	13.350	4711992	2.92	2481552	3.36	1.90	V	Pentadecane
7	14.513	14.467	14.558	5279518	3.27	2495058	3.38	2.12		Hexadecane
8	15.658	15.617	15.708	5571878	3.45	2759193	3.74	2.02		Heptadecane
9	16.746	16.700	16.792	5752403	3.56	2724437	3.69	2.11		Octadecane
10	17.783	17.742	17.833	5912684	3.66	2896915	3.93	2.04		Nonadecane
11	18.770	18.725	18.817	6270216	3.89	3013320	4.08	2.08		Eicosane
12	19.716	19.675	19.767	6319882	3.92	3101755	4.20	2.04		Heneicosane
13	20.619	20.575	20.667	6643251	4.12	3131124	4.24	2.12		Docosane
14	21.486	21.442	21.533	6883446	4.27	3316705	4.49	2.08		Tricosane
15	22.319	22.275	22.367	6945571	4.30	3370539	4.57	2.06		Tetracosane
16	23.119	23.075	23.167	7200127	4.46	3444442	4.67	2.09		Pentacosane
17	23.890	23.842	23.942	7212875	4.47	3340491	4.53	2.16		2-Methylpentacosane
18	24.633	24.592	24.717	8622347	5.34	3434730	4.65	2.51		Dotriacontane
19	25.350	25.308	25.400	7477266	4.63	3567811	4.83	2.10		Dotriacontane
20	26.043	26.000	26.092	7602121	4.71	3654106	4.95	2.08		Dotriacontane
21	26.714	26.667	26.767	7765248	4.81	3581381	4.85	2.17		Tetrapentacontane
22	27.376	27.333	27.417	7104917	4.40	3282898	4.45	2.16		Tetrapentacontane
23	28.096	28.050	28.150	7359257	4.56	2999462	4.06	2.45		Dotriacontane
24	28.899	28.850	28.958	7198798	4.46	2825615	3.83	2.55		Tritriacontane
25	29.809	29.758	29.875	6648334	4.12	2325660	3.15	2.86		Tetratriacontane
26	30.853	30.800	30.925	6383682	3.96	1962342	2.66	3.25		Pentatriacontane
				161359236	100.00	73797207	100.00			

Figure S31. Retention times of *n*-paraffins (C_{10}-C_{35}) analyzed under the same chromatographic conditions as the samples were used.

Table S1. Retention indices of Kovats (KIs) experimental and reported in the literature for the mixture of sterols.

Rt (min)	Compound identified	Main characteristic ions (*m*/*z*)	KIs	
			Exp.	Lit.
28.891	Campesterol (**5**)	43, 315, 400	3310	3305
29.173	Stigmasterol (**6**)	83, 394, 412	3324	3332
29.740	γ-sitosterol (**7**)	43, 303, 414	3351	3343
